# Exergames in the Rehabilitation of Burn Patients: A Systematic Review of Randomized Controlled Trials

**DOI:** 10.3390/ebj6040060

**Published:** 2025-11-27

**Authors:** Inês Santos, Marta Ferreira, Carla Sílvia Fernandes

**Affiliations:** 1 Abel Salazar Biomedical Sciences Institute, University of Porto, 4050-313 Porto, Portugal; inesfonsecasantos@hotmail.com; 2 Prelada Hospital, Burn Unit, 4250-448 Porto, Portugal; 3 INESC TEC, Institute of Systems and Computer Engineering, 4200-465 Porto, Portugal; mferreira@fe.up.pt; 4 Faculty of Engineering, University of Porto, 4200-465 Porto, Portugal; 5 School of Health, Polytechnic Institute of Viana do Castelo, 4900-314 Castelo, Portugal; 6 Aditgames, Association for Innovation, Technologies and Games in Health, 4490-565 Porto, Portugal

**Keywords:** burn patient, exergames, rehabilitation, technology

## Abstract

The rehabilitation of burn patients is essential and is intrinsically linked to conventional rehabilitation; the motivational challenges faced by burn patients in maintaining engagement with these rehabilitation programs are well known. It is understood that the use of other resources, particularly technological ones, associated with conventional rehabilitation could overcome these constraints and thereby optimize the rehabilitation program and health outcomes. The objective of this study is to synthesize the available evidence on the use of exergames in rehabilitation programs for burn patients. This systematic review was developed following the guidelines of the Joanna Briggs Institute (JBI). The search was conducted in the following databases: Medline^®^, CINAHL^®^, Sports Discus^®^, Cochrane^®^, and Scopus^®^ during May 2025. The PRISMA Checklist Model was used to organize the information from the selected studies. Seven RCTs were included, involving a total of 236 participants. Outcomes related to the use of exergames in the rehabilitation of burn patients were identified, including increased range of motion, functionality, strength, speed of movement, improved balance, reduced fear and pain, and satisfaction with the technological resource used. It is believed that the results of this review, which confirmed the advantage of using exergames, such as Nintendo Wii, PlayStation, Xbox Kinect, or Wii Fit, to optimize the functionality of burn patients, can support clinical decision-making and encourage the integration of exergames to improve rehabilitation programs for burn patients.

## 1. Introduction

Burns are recognized as some of the most painful and destructive injuries, both physically and psychologically, and the functional rehabilitation of burn patients presents many obstacles [1]. The healing process is often accompanied by complications, particularly contractures and hypertrophic scars, which highlights the importance of initiating rehabilitation programs as early as possible [2].

Rehabilitation is the primary process that supports the recovery or maintenance of functionality in burn patients; therefore, inadequate or delayed rehabilitation increases the likelihood of dysfunction [3]. Conventional therapy, which includes continuous and repetitive therapeutic exercises, as well as active and passive joint movements, although often unattractive, unengaging, and associated with unbearable pain, remains essential for the functional recovery of burn patients [4].

For this reason, it is understood that the use of new technological resources, such as exergames, may serve as valuable tools in the rehabilitation process of these patients. Exergames are a new generation of video games that require large body movements (involving the trunk and upper and/or lower limbs) within engaging digital environments [5]. Visual and auditory stimuli can be combined with various types of equipment (e.g., balance boards, immersion mats, dance mats, dumbbells, cameras, and other types of sensors and input devices) that require users to move to achieve a specific goal concept associated with gamification [6]. Exergames are advantageous because, beyond physiological benefits, they also promote psychological improvements related to entertainment and distraction [6].

Currently, there are several types of exergames, including those simulating aerobic exercises (e.g., walking, running) or sports (e.g., basketball, tennis, swimming, boxing, or dancing) [6]. Due to their accessibility, these technological resources have been progressively integrated into motor rehabilitation programs across different healthcare contexts, including those involving burn patients [7,8]. They are regarded as easily accessible technologies associated with enjoyable and motivating activities, increasingly complementing conventional rehabilitation because of their contribution to functional improvement [9]. For this reason, exergames are legitimately considered serious games for health, as their purpose extends beyond entertainment to achieving health-related outcomes [10]. Grounded in the conceptual framework of gamification, which advocates the use of game elements in non-game contexts, these resources have gained growing popularity due to their potential to enhance functional rehabilitation through interactivity that minimizes the perception of pain [11,12].

In other words, technological resources with gamified elements, when applied in healthcare contexts, facilitate engagement and patient participation in rehabilitation processes [13]. Recent studies suggest that the use of these resources, as an adjunct to conventional rehabilitation, can result in functional gains for burn patients [14]. One of the main benefits of exergames is their ability to incorporate functional movements comparable to those required in daily activities into the gameplay mechanics (e.g., combing hair, eating with utensils) [10].

The early implementation of rehabilitation programs for burn patients, justified by the need to prevent long-term complications, should begin during hospitalization and extend beyond clinical discharge [10]. However, pain, anxiety, and the lack of appeal of conventional rehabilitation programs remain major obstacles to adherence among burn patients [15]. To innovate and make this process more attractive, with lower dropout rates and greater functional gains, technology-based rehabilitation interventions have been developed, notably games—termed exergames because they combine exercise and play—such as Nintendo Wii [16].

In this context, it is essential to understand the true impact of integrating exergames into rehabilitation programs for burn patients. To provide a broader overview of outcomes associated with their use, this review includes studies involving both pediatric and adult populations, as the contexts of hospitalization, intended objectives, and main advantages of exergames are comparable across these groups. Therefore, this systematic review aims to synthesize the main findings reported in the scientific literature on this topic.

## 2. Materials and Methods

### 2.1. Study Design

The conduct of this systematic review followed the Joanna Briggs Institute (JBI) framework. The Preferred Reporting Items for Systematic Reviews and Meta-Analyses (PRISMA) checklist was also used to organize and systematize the methodology and the presentation of results [17]. The review protocol was registered in PROSPERO under ID 1073583. The authors attest that no Artificial Intelligence (AI) tools were used during the various stages of preparation of this article (conception, writing, and analysis), including in the interpretation of data and final writing.

### 2.2. Search Strategy

The research question was structured according to the PICO methodology (Population, Intervention, Comparator, Outcome) [17]. The population analyzed consisted of burn patients; the intervention was the implementation of rehabilitation programs with exergames; the comparator corresponded to conventional rehabilitation programs without the use of exergames; and the outcome was related to the impact of the intervention compared to the conventional method.

Randomized clinical trials were selected from the following databases: Medline^®^, CINAHL^®^, Sports Discus^®^, Cochrane^®^, and Scopus^®^, using indexed descriptors in each database associated with the study area. Boolean phrases were constructed using the AND and OR operators to achieve the research objectives (Table 1), and an additional search for studies was conducted by consulting the reference lists.

### 2.3. Eligibility Criteria

The inclusion criteria for this systematic review were (a) randomized clinical trials, (b) involving burn patients who were either hospitalized or undergoing home-based functional recovery, regardless of age, (c) with comparable pre- and post-intervention programs, (d) a control group with conventional rehabilitation programs, and (e) measurement of intervention outcomes. The exclusion criteria encompassed ineligible types of publications, such as abstracts, protocols, integrative review articles, editorials, commentaries, guidelines, and case studies. No restrictions were applied regarding the date of publication, to broaden the scope of studies relevant to the research question. The research was conducted between 1 May and 31 May 2025.

### 2.4. Study Selection

After searching and selecting the studies based on the inclusion and exclusion criteria, the articles were retrieved and imported into Rayyan Systems^®^, Inc. (Cambridge, MA, USA, Qatar Computing Research Institute) software, which facilitates the review process. After duplicate articles were removed, two reviewers screened the studies based on the title and abstract, selecting articles for full-text reading. A third reviewer participated in all stages of the process, ensuring confirmation of the eligibility of the included studies in cases where consensus was not reached.

### 2.5. Synthesis of Results

A descriptive presentation of the extracted data was performed, organized in a table, covering (a) general study information; (b) program objectives; (c) participant characteristics, such as number, gender, age, and burned body surface area; (d) description of the intervention, including the type of exergame, intervention objectives, program phase, frequency, intensity, intervention assessment, and outcomes; and (e) limitations.

Additionally, the possibility of conducting a meta-analysis of some outcomes was considered; however, due to the variability of the instruments used and the unavailability of certain data, even after contacting the authors, it was not possible to perform the meta-analysis.

### 2.6. Quality Assessment

The methodological quality of the included randomized clinical trials was assessed using the JBI Critical Appraisal Checklist for Randomized Controlled Trials [18]. It was determined that articles meeting at least 7 of the 13 criteria defined by the methodological quality assessment tool could be included in this systematic review [18].

## 3. Results

**Figure 1:**
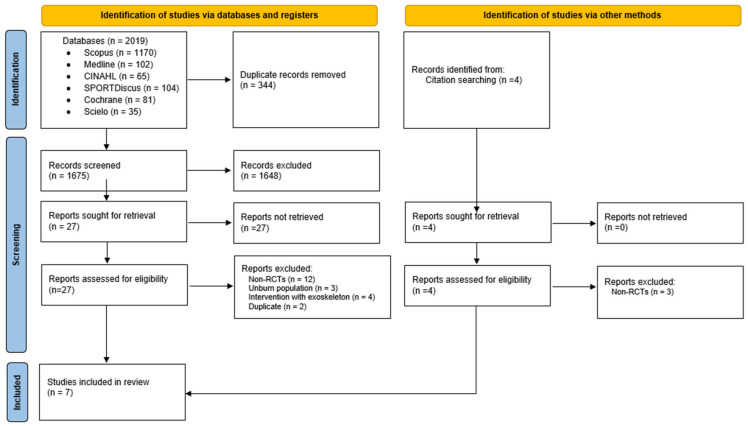
PRISMA flow diagram (2020).

Figure 1 presents the PRISMA flowchart, which schematically illustrates the research process that culminated in the selection of the seven RCTs [7,14,16,19,20,21,22] included in this review.

### 3.1. Studies Characteristics

The methodological quality of the studies was assessed according to predefined criteria, and no article was excluded since all scored above 7. Two studies met most of the methodological quality criteria (≥10/13), three studies met 8/13 criteria, one study achieved 9/13 criteria, and another met 11/13 criteria. These results, detailed in Table 2, should be considered when interpreting the reported effects.

### 3.2. Participants Characteristics

Table 3 presents the main characteristics of the participants (number, age, gender). There are significant variations among the study participants. Sample sizes ranged from 17 [20] to 66 participants [21]. In all studies, male participants were in the majority. Regarding the age range, three out of the seven studies included adult participants (18 to 78 years old) [7,14,19], another three included children between 5 and 17 years old [20,21,22], and only one study had a mixed age range, with participants between 16 and 59 years old [16].

### 3.3. Intervention Characteristics

The implemented rehabilitation programs differed from each other in terms of the phase of implementation (hospital or home setting) and, essentially, the frequency of use. Regarding the phase of program implementation, the analysis showed that it took place in hospital settings [16,19,21], in home settings after clinical discharge [14,22], and in a combined manner (hospital and home) [7,20]. The exergame intervention was carried out using well-known commercial interactive gaming platforms such as Nintendo Wii [16,19,22], Xbox Kinect [6,21], Playstation II Eye Toy [20], and Wii Fit [14].

The intensity of the rehabilitation programs was not described in most of the included studies [7,16,19,20,21], with only two studies [14,22] reporting it as being directly related to performance or progression within the interactive game itself. Regarding intervention frequency, protocols with daily or twice-daily sessions [7,16,20] were identified, as well as others with a frequency of 2 to 3 times per week [14,21,22], with session duration ranging from 15 to 35 min.

The total duration of the programs varied greatly, ranging from a minimum of 3 days [19] to a maximum of 6 months in a study that combined hospitalization and home setting [20]. In studies conducted exclusively in the hospital setting, the duration of the program was not specified, as it depended on the length of hospitalization [16,21], except for one study [19], where the protocol lasted only 3 days. Programs conducted in the home setting or in a mixed regimen lasted between 7 days [6] and 12 weeks [22]. It is also noteworthy that the implications of burn injuries extend beyond the hospital setting, as evidenced by the existence of studies conducted in mixed (hospital and home) settings [7,20], or exclusively at home [14,21], both in children and adults.

### 3.4. Outcomes

The objectives of the interventions analyzed in these studies targeted various outcomes for participants, including reduction in pain [7,16,19,20], fear [16], and anxiety [19] associated with rehabilitation treatments; promotion of joint range of motion [7,15,19,20,21,22], balance [14], muscle strength [14], and physical activity [7,21,22]; and assessment of participant satisfaction and engagement with the use of exergames [6,19].

The outcomes identified in the selected studies (Table 4) are related to different dimensions of burn pathophysiology, encompassing the physical, psychological, and behavioral implications for burn patients (Table 4). The main outcome assessed, present in four of the seven studies [7,16,19,20], was range of motion (ROM), usually measured with a goniometer.

Balance, a key indicator of functionality that reflects the relationship between health status and contextual factors, was assessed in only one study [14], using the Stability Index—Biodex Balance System and the Timed Up and Go Test. Pain was assessed in four studies [7,16,19,20] through the Visual Analogue Scale [16,19], pain scales adapted from previous virtual reality studies [20], and an analog pain scale [7]. The duration of data collection ranged from 3 days [19] to 6 months [20].

Anxiety and fear associated with the intervention were also evaluated [6,16,19], using different instruments—the Visual Analogue Scale adapted for anxiety, the Pain Anxiety Symptoms Scale, and the Tampa Scale for Kinesiophobia.

Regarding other physiological parameters, changes in heart rate and perceived exertion associated with exergame use were measured in one study [20], which recorded increased heart rate (bpm) and employed a perceived exertion scale ranging from 0 (no fatigue) to 10 (extreme fatigue). Muscle strength was assessed in only one study [14], using isokinetic dynamometry of the quadriceps and hamstrings.

From a functional perspective, several assessment tools were applied [7,14,21,22], including the High-Level Mobility Assessment Tool [14], Lower Limb Functional Index [14], 6-Minute Walk Test [14], Jebsen Hand Function Test [22], Valpar 9 Whole-Body Range of Motion Work Sample Test [19], and Disabilities of the Arm, Shoulder and Hand [7]. Data for these outcomes were collected over periods ranging from 3 days [19] to 12 weeks [14]. Other outcomes included movement speed and duration [21], and satisfaction [7,16,19,20], which was assessed through self-reports, therapist reports, subjective questionnaires, the Wong–Baker Faces, and an adapted Visual Analogue Scale, where 0 represents “not satisfied at all” and 10 “completely satisfied”.

Adherence to rehabilitation programs using exergames was remarkable and sustained in all studies, and no adverse events, either psychological or hemodynamic, were identified in any of the studies.

The analysis of the results presented in Table 4 demonstrates that the use of exergames in rehabilitation programs leads to improvements in various clinical and functional outcomes when compared to conventional rehabilitation [7,14,16,19,20,21,22]. Statistically significant differences were observed, particularly in joint range of motion (ROM) [7,16,19,20,21], balance and mobility [14], muscle strength [14], functional capacity [14,21,22] and movement speed [21]. Improvements in satisfaction, adherence, and participant engagement indicators were also reported [7,14,19,20]. In some studies, gains in movement speed and quality were also observed, reinforcing the potential of exergames to promote functional autonomy [21].

A trend towards reduced levels of pain, anxiety, and fear during rehabilitation was also observed [7,16,19,20].

## 4. Discussion

The main objective of this systematic review was to synthesize the available evidence on the effectiveness of exergames in rehabilitation programs for burn patients, integrating the findings of randomized clinical trials published to date.

The limited number of studies identified reflects the emerging nature of this research area in this specific population, in contrast to other fields of rehabilitation where the use of exergames has already been more extensively investigated [23,24].

Among the seven included studies, there was not only considerable geographical and methodological variability but also the inclusion of different age groups and varying levels of injury severity, encompassing both pediatric and adult populations [7,14,16,19,20,21,22]. Furthermore, the interventions themselves were highly heterogeneous, with reported frequency, intensity, and duration ranging widely (from several days to up to 6 months of follow-up). These particularities limit the generalizability of the results, which should therefore be interpreted with due caution. In this review, it was observed that different exergame devices, such as Wii Fit [14], Nintendo Wii [16,19,20,21], and Xbox Kinect [7,22], were employed in the randomized clinical trials analyzed. Although these devices were not originally designed for rehabilitation purposes, their practicality, ease of use, playful potential, and capacity to promote physical activity render them adaptable and effective tools in clinical settings [13,19,25,26]. This adaptability, together with the accessibility and user familiarity with these technologies, has contributed to their progressive integration into rehabilitation programs [23,25,26].

Regarding pain, fear, and anxiety, although the results did not reach statistical significance in most studies [7,16,19,20], there was a tendency toward reduction, suggesting a positive impact of exergames not only on the physical level but also on the psychological and emotional levels. This trend is consistent with other studies, which highlight the role of exergames and other digital technologies, such as virtual reality, in promoting pain distraction [26,27]. The distraction effect associated with the use of these resources allows for the optimization of rehabilitation, impacting the improvement or maintenance of associated functional outcomes, such as range of motion, as also demonstrated by the results of our study [26,28,29].

The fear associated with pain, or fear of movement, which is intrinsically linked to the rehabilitation process, has been evaluated in several studies [5,7,16,20]. This concept, applied in various healthcare contexts, particularly in chronic pain and immediate postoperative situations, is also observed in burn patients due to the multiple pain experiences they endure [7,16]. Different types of pain related to burns contribute to kinesiophobia: burn pain, musculoskeletal immobilization pain resulting from burns, acute pain occurring suddenly during hospitalization, pain following surgical procedures, pain during dressing changes, and pain associated with rehabilitation processes [5]. These painful experiences foster anxiety and fear, reinforcing beliefs and behaviors that avoid movement [5,7,16,20]. Although the results were not statistically significant, they indicate a trend toward reduced fear of movement in participants who engaged with exergames [7,16,20]. This reduction in fear may facilitate adherence to and engagement in rehabilitation programs, thereby enhancing functional outcomes [5,7,16,20].

Regarding satisfaction, this outcome proved particularly relevant, with statistically significant results identified in multiple studies [6,13,18,19]. These findings suggest that the integration of exergames into rehabilitation programs for burn patients contributes to greater participant satisfaction, resulting in a more motivating and positive rehabilitation experience.

In summary, the available evidence supports that the integration of exergames into rehabilitation programs can enhance functional gains and treatment adherence, representing an effective strategy for the rehabilitation of burn patients [6,13,15,18,19,20,21].

## 5. Implications for Practice and Research

The results of this study have essential clinical and research implications for the implementation of rehabilitation programs for burn patients. From a clinical perspective, the results of this systematic review reinforce the importance and advantages of including exergames in rehabilitation programs to increase adherence, motivation, and satisfaction with such programs [6,13,15,18,19,20,21]. When combined with conventional rehabilitation, exergames are important tools for optimizing and maintaining functionality in burn patients [7,14,16,19,20].

The findings indicate that, from the point of view of the requirements for eventual implementation, the need for funding to purchase the necessary devices may be a challenge and an institutional constraint [23,25]. On the other hand, adequate supervision by the clinical team is necessary to ensure proper use of the systems and optimization of resources as part of the hospitalization process [26]. This supervision requires adequate training of the clinical team responsible for implementing the rehabilitation program, regarding infection control, since it requires proper sanitization of the devices between users (subject to hospitalization in isolation due to the nature of the burn pathophysiology and susceptibility to infection) [27,28]. The use of these devices may also be conditioned by resistance to the adoption of new technologies on the part of the clinical team and/or users and by the constant development/updating of systems [23,26].

In terms of opportunities for future research, it would be pertinent to invest in the development of exergames specifically designed to meet the needs of burn patients, considering the physical, sensory, and psychological limitations inherent to this condition [25,29].

## 6. Limitations

The main limitations of this systematic review are related to the variability in sample size and participant characteristics, as well as the significant heterogeneity of the instruments used to assess outcomes associated with the use of exergames in the rehabilitation of burn patients, which precluded the performance of a meta-analysis. The methodological diversity of the studies analyzed, including differences in intervention protocols (types of exergames, duration, frequency, and context of application), further limits the generalizability of the results.

In addition, potential bias may have been introduced by the high heterogeneity among the included studies, particularly in terms of interventions applied, populations studied, and body areas affected. This diversity hinders direct comparison of the results and may have influenced how the findings were synthesized and interpreted, despite adherence to JBI/PRISMA guidelines and independent review procedures.

Future studies involving larger samples and similar pathophysiological conditions are needed to enable the standardization of rehabilitation protocols, particularly regarding the intensity, frequency, and duration of the different components of the programs.

## 7. Conclusions

This systematic review and its preliminary findings demonstrate that the integration of exergames into rehabilitation programs for burn patients constitutes a promising approach. Despite the heterogeneity of the included studies and the methodological limitations identified, significant gains were observed, particularly in terms of patient motivation, adherence, and engagement in the rehabilitation process, with no adverse effects associated with their use.

However, given the preliminary stage of research in this field, caution should be exercised when generalizing the results, as further investigations with larger sample sizes and standardized protocols are required to strengthen the evidence base and support robust recommendations for clinical practice.

## Figures and Tables

**Table 1:** Search strategy according to the selected database.

Database	Research Strategy
Medline	((MH “Burns”) OR (MH “Burn Units”) OR (“Burn”) OR (“Burn Unit”) OR (“Burn Patient*”)) AND ((MH “Exergaming”) OR (MH “Gamification”) OR (MH “Video Games”) OR (“Exergaming”) OR (“Gamification”) OR (“Exergame”) OR (“Wii”) OR (“Nintendo”) OR (“Xbox”) OR (“Playstation”) OR (“video games”) OR (“Game”) OR (“Gamming”) OR (“Gamification”) OR (“Serious Game”) OR (“Gamified”))
CINAHL	((MH “Burns”) OR (MH “Burn Units”) OR (“Burn”) OR (“Burn Unit”) OR (“Burn Patient *”)) AND ((MH “Exergaming”) OR (MH “Gamification”) OR (MH “Video Games”) OR (“Exergaming”) OR (“Gamification”) OR (“Exergame”) OR (“Wii”) OR (“Nintendo”) OR (“Xbox”) OR (“Playstation”) OR (“video games”) OR (“Game”) OR (“Gamming”) OR (“Gamification”) OR (“Serious Game”) OR (“Gamified”))
Scopus	((“Burn*” or “Burn Unit*” or “Burn Patient *”)) AND ((“Exergaming” or “Gamification” or “Exergame *” or “Wii” or “Nintendo” or “Xbox” or “Playstation” or “Video Games” or “Game” or “Gaming” or “Serious game” or “Gamified”))
SciELO	((“Burn*” or “Burn Unit *” or “Burn Patient*”)) AND ((“Exergaming” or “Gamification” or “Exergame *” or “Wii” or “Nintendo” or “Xbox” or “Playstation” or “Video Games” or “Game” or “Gaming” or “Serious game” or “Gamified”))
Cochrane	((“Burn*” or “Burn Unit *” or “Burn Patient *”)) AND ((“Exergaming” or “Gamification” or “Exergame *” or “Wii” or “Nintendo” or “Xbox” or “Playstation” or “Video Games” or “Game” or “Gaming” or “Serious game” or “Gamified”))
Sports Discus	((DE “Burns”) OR (DE “Burn Units”) OR (“Burn”) OR (“Burn Unit”) OR (“Burn Patient *”)) AND ((DE “Exergame”) OR (“Gamification”) OR (“Video Games”) OR (“Exergaming”) OR (“Gamification”) OR (“Exergame”) OR (“Wii”) OR (“Nintendo”) OR (“Xbox”) OR (“Playstation”) OR (“video games”) OR (“Game”) OR (“Gamming”) OR (“Gamification”) OR (“Serious Game”) OR (“Gamified”))
	Legend: * Represents truncation to include different word endings in the search strategy

**Table 2:** Methodological assessment of the studies included in the systematic review.

Study	Criteria													Total
	C1	C2	C3	C4	C5	C6	C7	C8	C9	C10	C11	C12	C13	
Yohannan et al., (2011) [19]	N	N	Y	N	N	Y	Y	Y	Y	Y	Y	Y	U	8/13
Parker et al., (2016) [16]	Y	N	Y	N	N	U	Y	Y	Y	Y	Y	Y	Y	9/13
Parry et al., (2015) [17]	Y	N	Y	N	N	U	Y	U	Y	Y	Y	Y	Y	8/13
Voon et al., (2016) [8]	Y	Y	Y	N	N	Y	Y	Y	Y	Y	Y	Y	Y	11/13
Lozano et al., (2018) [21]	N	N	Y	N	N	N	Y	Y	Y	Y	Y	Y	Y	8/13
Radwan et al., (2020) [22]	Y	N	Y	N	N	Y	Y	Y	Y	Y	Y	Y	Y	10/13
Basha et al., (2022) [14]	Y	N	Y	N	N	Y	Y	Y	Y	Y	Y	Y	Y	10/13

Legend: Y = yes; N = no; U = unclear.

**Table 3:** Characteristics of the included studies.

Author, Year, Country	Study Objective	Participants					Intervention					Instruments	Limitations
		N	Gender	Age	TBSA	Body Part Injured	Exergame	Aim	Phase	Frequency	Intensity		
[19] Yohannan et al., 2011, USA	To evaluate the feasibility and effects of Nintendo Wii–based rehabilitation in patients with acute burns	23	10 F 13 M	20–78	0.5–23%	Wrist, elbow, shoulder, hips, knees and ankles	Nintendo Wii	Pain, anxiety, range of motion, functionality, satisfaction, sense of presence	Inpatient	15 min, 3 sessions in 3 consecutive days	Not described	Presence, Enjoyment, VAS, Anxiety, AROM, Valpar 9 Whole Body Range of motion, Work Sample Test	Learning curve not considered; Wii not optimized for rehabilitation; animations and small screen limited user experience
[16] Parker et al., 2016, Australia	To evaluate the feasibility of Nintendo Wii as an adjuvant in the rehabilitation of patients with severe burns	22	5 F 17 M	16–59	0.5–10%	Upper and lower limbs	Nintendo Wii	Pain, fear, range of motion	Inpatient	20–30 min, 2 times a day for 5 days	Not described	VAS, PASS, ROM	No limitations related to the use of the exergame are described
[20] Parry et al., 2015, USA	To compare the results of conventional therapy and video game therapy in pediatric patients	17	3 F 14 M	5–18	Mean: 48%	Axilla, shoulder	Playstation II Eye Toy (PE)	Range of motion, pain	Inpatient and outpatient	25 to 35 min, 2 times a day, 5 days a week for 3 weeks (hospitalization) + 6 months (outpatient)	Progression and intensity were based on game scores	ROM, VAS	No limitations related to the use of the exergame are described
[7] Voon et al., 2016, Australia	To compare the results of conventional therapy and Xbox Kinect -based rehabilitation as adjuvant	30	11 F 19 M	23–40	1.5–7%	Shoulder, arm, wrist and hand	Xbox Kinect	Physical activity, functionality, pain	Inpatient and outpatient	15 min, 2 times a day for 7 days	Not described	Rehabilitation activity, QuickDASH, VAS, TAMPA	Dressings hindered device detection; intervention time overestimated; ROM not monitored; sensor range limited use in bedridden patients
[21] Lozano et al., 2018, South Africa	To investigate the effect of using Xbox Kinect as an adjuvant to physical therapy after hospital discharge	66	29 F 37 M	5–9	4.5–16%	Head, neck, upper and lower limbs, trunk, buttocks and genitals	Xbox Kinect	Range of motion, physical activity, enjoyment, satisfaction	Inpatient	15 to 30 min, 2 times a sessions (7 to 20 sessions)	Not described	ROM, ASK©p scores, Fun and enjoyment	No limitations related to the use of the exergame are described
[22] Radwan et al., 2020, Egypt	To compare the effect of Nintendo Wii intervention with conventional rehabilitation in burned children	44	17 F 27 M	7–12	4–9%	Hand, shoulder, face, arm, forearm and wrist	Nintendo Wii	Spatiotemporal parameters, functionality	Outpatient	30 min, 3 times a week for 6 weeks	Not described	spatiotemporal parameters, JHFT	Intervention with Nintendo Wii must be carried out under supervision
[14] Basha et al., 2022, Egypt	Improve functionality, mobility, exercise capacity muscle, strength and balance with Wii Fit based rehabilitation	34	9 F 25 M	18–40	>40%	Lower limbs	Wii Fit	Strength, aerobic, and balance	Outpatient	30 min, 3 days a week for 12 weeks	Progression and intensity were based on game scores	HiMAT, LLFI, 6MWT, quadriceps and hamstring strength, stability index and TUG	Three-dimensional analysis of the movement responsible for walking not allowed

Legend: M—male; F—female; TBSA—total body surface area (%); VAS—Visual Analogue Scale; PASS—Pain Anxiety Symptoms Scale; ROM—Range of Motion; AROM—Active Range of Motion; QuickDASH—Quick Disabilities of the Arm, Shoulder and Hand; TAMPA—Tampa Scale for Kinesiophobia; ASK©p scores—Activities Scale for Kids©p; JHFT—Jebsen Hand Function Test; HiMAT—High-Level Mobility Assessment Tool; LLFI—Lower Limb Functional Index; 6MWT—Six-Minute Walk Test; TUG—Timed Up and Go.

**Table 4:** Main outcomes of the included studies.

Outcome	Instrument	Reference	Control Group (Mean)	Intervention Group (Mean)	*p* Value
ROM	Goniometry Shoulder flexion (hand to head)	Parry et al., 2015 [20]	7.6	10.2	<0.001
	Goniometry Shoulder abduction (hand to head)	Parry et al., 2015 [20]	2.4	1.9	<0.001
	Goniometry Neck flexion (hand to head)	Parry et al., 2015 [20]	2.9	−2.3	0.009
	Goniometry Elbow flexion (hand to head)	Parry et al., 2015 [20]	3.8	2.2	0.004
	Goniometry Shoulder flexion (High reach)	Parry et al., 2015 [20]	16.4	9.3	0.04
	Goniometry Shoulder abduction (High reach)	Parry et al., 2015 [20]	−16.4	−4.4	0.36
	Goniometry Elbow extension (High reach)	Parry et al., 2015 [20]	9.8	1.2	0.51
	Goniometry Shoulder flexion (wave)	Parry et al., 2015 [20]	8.5	6.0	0.27
	Goniometry Shoulder abduction (wave)	Parry et al., 2015 [20]	−4.43	−1.7	0.36
	Goniometry Shoulder ext rot (wave)	Parry et al., 2015 [20]	−6.9	−3.5	0.152
	Goniometry	Yohannan et al., 2011 [19]	1.71	2.26	0.81
	Goniometry	Lozano et al., 2018 [21]	15.3	18.8	<0.01
	Goniometry Shoulder abduction	Parker et al., 2016 [16]	0	1	0.94
	Goniometry Elbow flexion	Parker et al., 2016 [16]	0	−2.5	>1
	Goniometry Elbow extension	Parker et al., 2016 [16]	0	0	>1
	Goniometry Wrist flexion	Parker et al., 2016 [16]	15	−1.5	>1
	Goniometry Wrist extension	Parker et al., 2016 [16]	−1.5	−1	>1
	Goniometry Hand span (cm)	Parker et al., 2016 [16]	0.5	1.5	>1
	Goniometry Pulp to distal palmar crease (cm)	Parker et al., 2016 [16]	0	0	>1
	Goniometry Knee flexion	Parker et al., 2016 [16]	14	−8	>1
	Goniometry Knee extension	Parker et al., 2016 [16]	0	0	>1
	Goniometry Ankle dorsiflexion	Parker et al., 2016 [16]	4	10	>1
	Goniometry Plantar Flexion	Parker et al., 2016 [16]	5	−5	>1
Balance	SI	Basha et al., 2022 [14]	3.92	2.35	0.0006
	TUG	Basha et al., 2022 [14]	12.71	7.82	0.0003
Pain	Pain scale	Parry et al., 2015 [20]	+0.18	+0.047	0.02
	VAS	Yohannan et al., 2011 [19]	0.65	0.32	0.07
	APS	Voon et al., 2016 [7]	+0.73	+0.30	0.111
Fear	PASS	Parker et al., 2016 [16]	−4.5	−12	>1
	TAMPA	Voon et al., 2016 [7]	37.4	36.9	0.754
Heart rate and perceived exertion	Heart Rate	Parry et al., 2015 [20]	8.4	13.5	0.91
	Perceived exertion	Parry et al., 2015 [20]	3.2	3.9	0.41
Satisfaction	Enjoyment and presence	Yohannan et al., 2011 [19]	0.30	0.39	0.73
	Satisfaction index	Voon et al., 2016 [7]	7.8	8.53	<0.0001
	Fun and Enjoyment	Lozano et al., 2018 [21]	3	5	<0.01
	Compliance (mean)	Parry et al., 2015 [20]	85%	90%	0.43
	Compliance	Voon et al., 2016 [7]	26.7	49.37	<0.001
Muscle strength	Quadriceps strength	Basha et al., 2022 [14]	66.18	75.59	0.0001
	Hamstring strength	Basha et al., 2022 [14]	57.76	65.06	0.001
Anxiety	VAS	Yohannan et al., 2011 [19]	0.23	0.12	0.77
Function	HiMAT	Basha et al., 2022 [14]	39.29	46.29	0.001
	LLFI	Basha et al., 2022 [14]	63.59	74.94	0.0005
	6MWT	Basha et al., 2022 [14]	387.29	460.35	0.0004
	JHFT	Radwan et al., 2020 [22]	−3.91	−9.87	<0.001
	Valpar 9 Whole	Yohannan et al., 2011 [19]	- 0.74	−1.12	0.43
	QuickDASH	Voon et al., 2016 [7]	43.7	38	0.754
Velocity and whole of movement	Whole movement duration—Hand to Head	Radwan et al., 2020 [22]	−0.03	−0.1	<0.001
	Whole movement duration—Hand to Mouth	Radwan et al., 2020 [22]	−0.05	−0.09	<0.001
	Whole movement duration—Hand to Contralateral Shoulder	Radwan et al., 2020 [22]	−0.06	−0.13	<0.001
	Peak velocity—Hand to Head	Radwan et al., 2020 [22]	0.02	0.34	<0.001
	Peak velocity—Hand to Mouth	Radwan et al., 2020 [22]	0.16	0.27	<0.001
	Peak velocity—Hand to Contralateral Shoulder	Radwan et al., 2020 [22]	0.13	0.3	<0.001
	Time to peak velocity—Hand to Head	Radwan et al., 2020 [22]	−1.9	−15.38	<0.001
	Time to peak velocity—Hand to Mouth	Radwan et al., 2020 [22]	−12.27	−23.75	<0.001
	Time to peak velocity—Hand to Contralateral Shoulder	Radwan et al., 2020 [22]	−21.15	−29.01	<0.001

Legend: 6MWT—6-Minute Walk Test; APS—analog pain scale; Compliance—self report; Compliance (mean)—self report; Enjoyment and engagement—reports by the therapists; Enjoyment and presence—subjective rating questionnaires; Fun and Enjoyment—Wong–Baker Faces; Heart rate—increase in beats per minute; HiMAT—High-level Mobility Assessment Tool; JHFT—Jebsen Hand Function Test; LLFI—Lower Limb Functional Index (LLFI); Pain scale (0–10)—adapted from previous studies on virtual reality (VR); PASS—Pain Anxiety Symptoms Scale; Perceived exertion—scale of 0 to 10; QuickDASH—Disabilities of the Arm, Shoulder and Hand; ROM—range of motion; Satisfaction index—VAS 1–10; SI—Stability Index—Biodex Balance System; TAMPA—Tampa scale for kinesiophobia; TUG—Timed Up and Go Test; Valpar 9 Whole—Body Range of Motion Work Sample Test; VAS—Visual Analogue Scale (0–10).

## Data Availability

The data presented in this study is available on request from the corresponding author.

## Abbreviations

6MWT	6-Minute Walk Test
APS	Analog Pain Scale
AROM	Active Range of Motion
ASK©p	Activities Scale for Kids©p
F	Female
HiMAT	High-Level Mobility Assessment Tool
JHFT	Jebsen Hand Function Test
LLFI	Lower Limb Functional Index
M	Male
PASS	Pain Anxiety Symptoms Scale
QuickDASH	Quick Disabilities of the Arm, Shoulder and Hand
ROM	Range of Motion
SI	Stability Index (Biodex Balance System)
TAMPA	Tampa Scale for Kinesiophobia
TBSA	Total Body Surface Area
TUG	Timed Up and Go Test
VAS	Visual Analogue Scale
